# Induction of Acquired Tolerance Through Gradual Progression of Drought Is the Key for Maintenance of Spikelet Fertility and Yield in Rice Under Semi-irrigated Aerobic Conditions

**DOI:** 10.3389/fpls.2020.632919

**Published:** 2021-02-18

**Authors:** V. S. Lekshmy, Preethi Vijayaraghavareddy, A. N. Nagashree, Vemanna S. Ramu, Venkategowda Ramegowda, Udayakumar Makarla, Sheshshayee Sreeman

**Affiliations:** ^1^Department of Crop Physiology, University of Agricultural Sciences, Bengaluru, India; ^2^Department of Plant Sciences, Centre for Crop Systems Analysis, Wageningen University & Research, Wageningen, Netherlands; ^3^Regional Centre for Biotechnology, Faridabad, India

**Keywords:** acquired tolerance traits, reactive oxygen species, gradual stress, rice, reactive carbonyls

## Abstract

Plants have evolved several adaptive mechanisms to cope with water-limited conditions. While most of them are through constitutive traits, certain “acquired tolerance” traits also provide significant improvement in drought adaptation. Most abiotic stresses, especially drought, show a gradual progression of stress and hence provide an opportunity to upregulate specific protective mechanisms collectively referred to as “acquired tolerance” traits. Here, we demonstrate a significant genetic variability in acquired tolerance traits among rice germplasm accessions after standardizing a novel gradual stress progress protocol. Two contrasting genotypes, BPT 5204 (drought susceptible) and AC 39000 (tolerant), were used to standardize methodology for capturing acquired tolerance traits at seedling phase. Seedlings exposed to gradual progression of stress showed higher recovery with low free radical accumulation in both the genotypes compared to rapid stress. Further, the gradual stress progression protocol was used to examine the role of acquired tolerance at flowering phase using a set of 17 diverse rice genotypes. Significant diversity in free radical production and scavenging was observed among these genotypes. Association of these parameters with yield attributes showed that genotypes that managed free radical levels in cells were able to maintain high spikelet fertility and hence yield under stress. This study, besides emphasizing the importance of acquired tolerance, explains a high throughput phenotyping approach that significantly overcomes methodological constraints in assessing genetic variability in this important drought adaptive mechanism.

## Introduction

Rice (*Oryza sativa* L.), the most important staple cereal, accounts for more than half the water used in agriculture and represents a highly unsustainable practice in the face of climate change and water shortages (Basha et al., 2016; Kogan et al., 2019). Thus, cultivating rice with low water input to save water is quite essential, and it can only happen when the water requirement for a given yield potential is reduced (Foley et al., 2011). A strategic selection of relevant physiological traits for crop improvement and an appropriate agronomy of rice cultivation are needed to achieve the combined goal of sustaining productivity and saving irrigation water (Lambrecht et al., 2016; Sheshshayee et al., 2018). Semi-irrigated aerobic cultivation is one among several promising water-saving practices developed (Bouman et al., 2005; Nie et al., 2012). This method, with its periodic surface irrigation, has been shown to save around 50% of irrigation water. However, a water-limiting situation arises between episodes of irrigation, which reduces the yield potential despite the water saving advantage (Fereres and Soriano, 2007). Therefore, evolving appropriate rice cultivars well-suited to the aerobic conditions and with a sustained, if not increased, productivity is the most important need of the day.

Significant progress has been made in identifying specific relevant physiological traits that impart drought tolerance for improving productivity under water-limited conditions. The relevance of root traits (Abd Allah et al., 2010; Comas et al., 2013; Uga et al., 2015), water use efficiency (Richards et al., 2002; Sheshshayee et al., 2003; Blum, 2017; Dey et al., 2019), radiation use efficiency (Adeboye et al., 2016; Hao et al., 2016), etc. have been well established. However, lack of high throughput and accurate phenotyping options has often been quoted as one of the major reasons for the low pace of adoption of breeding for physiological traits. Furthermore, a comprehensive improvement in drought adaptation is possible only when several relevant traits are combined into the same genotype (Raju et al., 2014; Sheshshayee et al., 2018; Dharmappa et al., 2019). Three categories of traits operating at morphological, physiological, and cellular level would be needed for a comprehensive improvement in drought adaptation (Collins et al., 2008; Sheshshayee et al., 2018). While morphological and physiological traits such as canopy and root architecture, metabolic rates, biomass partitioning, reproductive growth, phenology etc. are constitutive and integral in nature (Haling et al., 2010; Sheshshayee et al., 2018), plants require several cellular level processes to maintain tissue turgor as well as metabolism collectively referred to as “adaptive” traits or “acquired tolerance” traits (Collins et al., 2008; Vijayaraghavareddy et al., 2020a). Most of the recent trait-based breeding efforts have so far attempted to improve or introgress constitutive traits. Although this strategy has great promise, combining these with acquired tolerance has greater relevance in achieving a comprehensive improvement in drought adaptation as well as productivity under water-limiting conditions.

Unlike constitutive traits, the acquired tolerance traits are expressed only upon exposure to stress (Sung et al., 2003; Senthil-kumar et al., 2007). Abiotic stresses, like soil moisture stress, develops progressively, and the rate of stress development generally depends on the prevailing weather conditions and soil texture (Ferrante and Mariani, 2018). Thus, plants initially experience a milder stress level, which can trigger the expression of acquired tolerance traits. This phenomenon would be extremely important, especially under rain-fed conditions. It is also known that significant genetic variability exists in the ability to respond to mild stress among several crop species (Clauw et al., 2016). That is, a genotype with greater propensity to respond to mild stress would have a higher level of adaptability subsequently if the stress level gets severe. Raju et al. (2014) demonstrated that genotypes with higher acquired tolerance in the background of sufficiently high constitutive traits showed a better field level adaptation.

Having recognized the importance of acquired tolerance traits, significant global initiatives led to the enumeration of several mechanisms and processes that govern them. Among several mechanisms, preventing the generation and scavenging the reactive oxygen species (ROS) and reactive carbonyl compounds (RCCs) appear to be the most prominent and relevant acquired tolerance response (Nisarga et al., 2017; Vemanna et al., 2020). Although under normal conditions also a low to moderate level of ROS and RCCs would be needed to maintain cellular signaling (Zhang et al., 2016), an imbalance between the production of ROS, RCCs, and their scavenging through antioxidant systems take center stage that determines the tolerance or susceptibility of a plant to stress (McCord, 2000; Vemanna et al., 2020). The ROS and RCC scavenging mechanisms are significantly upregulated when stress is mild, which protect cellular metabolism when stress becomes severe or lethal (Kumar et al., 1999; Senthil-kumar et al., 2003; Vidya et al., 2017). Based on the hypothesis that plants get primed during exposure to milder stress levels, a temperature induction response (TIR) technique was developed using germinated seedlings (Uma et al., 1995; Senthil-kumar et al., 2003; Vidya et al., 2017). However, capturing the variability in drought induction response has remained a challenge. The novel drought-simulator phenomics platform established at the Department of Crop Physiology, University of Agricultural Sciences, Bengaluru, India, with its automated transpiration-interfaced irrigation compliance, is capable of creating and maintaining a specific soil moisture status in the soil (Vijayaraghavareddy et al., 2020a).

Although these findings helped to understand acquired tolerance, lack of high throughput phenotyping made it difficult to validate this approach in different physiological phases of plants. Here, we standardize the methodology for capturing acquired tolerance traits at seedling phase in two contrasting genotypes of rice for drought using high throughput phenomics facility. Further, the role of oxidative stress management strategies associated with ROS and RCC scavenging mechanisms was examined as a potential measure of acquired tolerance in rice. We demonstrate a significant genetic variability in the acquired tolerance response to a gradually imposed stress protocol. Genotypes with superior acquired tolerance capacity maintain higher spikelet fertility and, hence, grain yields under drought stress.

## Materials and Methods

### Plant Material and Stress Treatment

Maintaining similar moisture status for all genotypes, irrespective of differences in their transpiration rates, is a prerequisite for the phenotyping of acquired tolerance traits. To evaluate the acquired tolerance mechanisms in rice, two experiments were conducted at the drought-simulator phenomics facility established at the Department of Crop Physiology, University of Agricultural Sciences, Bengaluru, India (12°58′N, 77°35′E). The first experiment was to capture stress response of rapid and gradually stressed plants using two well-characterized contrasting rice genotypes for drought stress response, viz., drought-sensitive BPT 5204 and tolerant AC 39000 (Pushpa, 2016). In the second experiment, the drought responses and acquired tolerance were screened among 17 diverse genotypes at flowering phase.

### Drought Imposition Protocol

The mini-lysimeter drought stimulator facility established at our center is designed to create gradual stress levels through the transpiration-interfaced automated irrigation system that mimics the natural environment condition. The facility is capable of gravimetrically determining the evapotranspiration of pot grown plants on a “real-time” basis. The automated irrigation system replenishes accurate volumes of water as and when the moisture status of soil decreases to 1.5% of the set field capacity (FC). A user-defined “dry-down” protocol ensures stress progression to mimic a real field-like situation (Vijayaraghavareddy et al., 2020a). The most prominent feature of this facility is its ability to ensure exactly the same level of soil moisture regime for plants irrespective of differences in leaf area and transpiration. Hence, this facility is most suited to capture acquired mechanisms at different growth phases.

## Experiment 1: Stress Imposition at Seedling Stage in Contrasting Rice Genotypes

Stress imposition was initiated on the 15th day after sowing for the two contrasting rice genotypes. Containers of 10-L capacity were filled with rooting mixture [red sandy loam soil and farm yard manure (FYM) in 3:1 ratio] were used for this study. Three treatments were created using phenomics platform:

1. Control: Plants were maintained at 100% FC throughout the experiment.

2. Rapid stress (RS): Rapid drought imposition was achieved by cessation of watering until the soil moisture reached to 50% FC.

3. Gradual stress (GS): The user defined protocol was tuned to reduce 5% of soil moisture per day.

**Figure 1:**
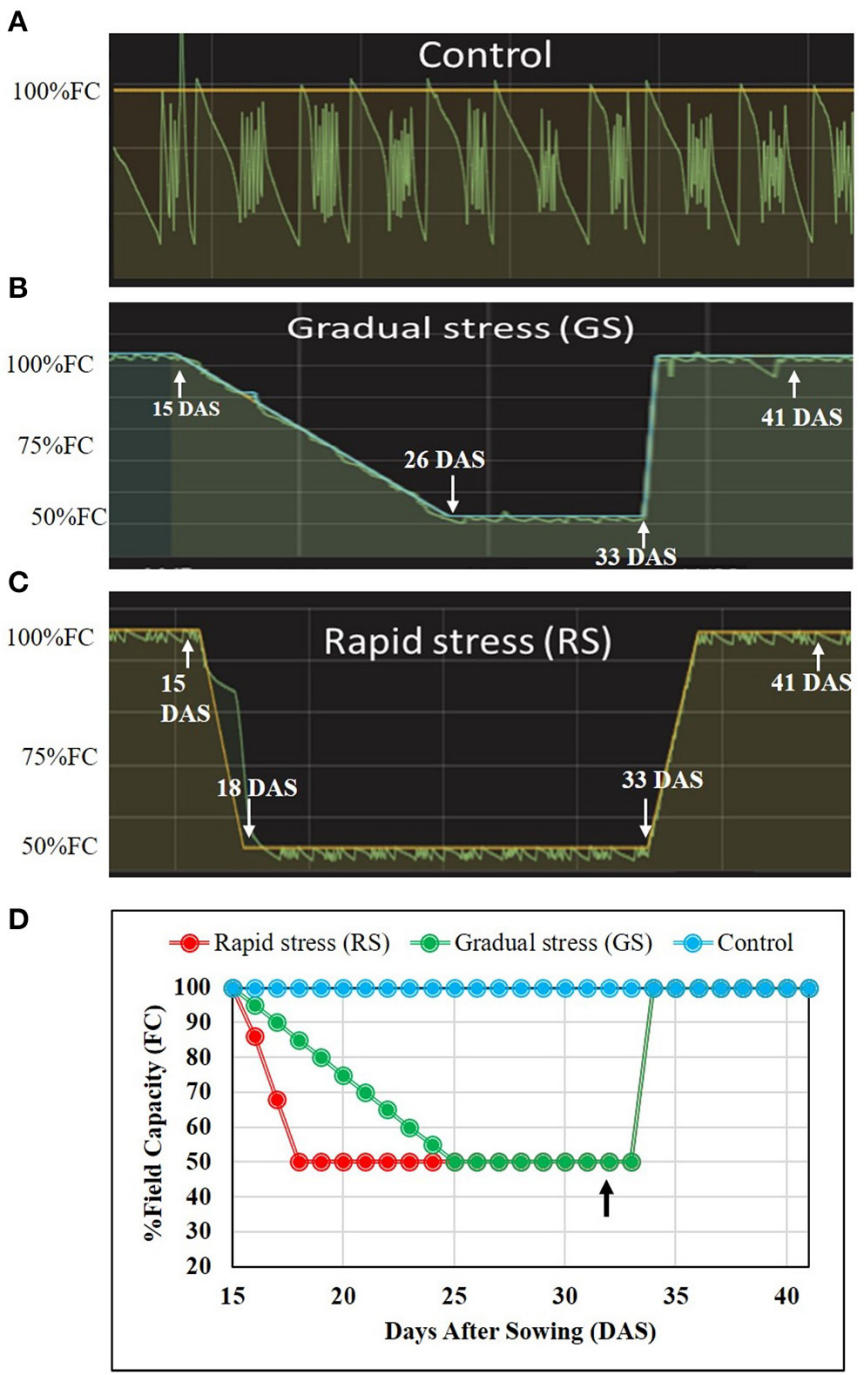
Graphical representation of the dry down protocol for stress imposing protocol in two rice genotypes. Fifteen-day-old plants were subjected to drought stress using the transpiration interfaced mini-lysimeter facility. **(A)** Control, plants maintained at 100% field capacity (FC) throughout the experiment. **(B)** The gradual stress using the dry-down protocol with 5% reduction in FC per day to achieve 50% FC. **(C)** Rapidly stressed plants where stress reaches to 50% FC within 3 days. The soil moisture was elevated to 100% FC at 33 days after sowing (DAS). **(D)** Representation of all the three different treatments, with an arrow indicating point at which samples were collected from all the three treatments.

After reaching 50% FC, both gradual and rapid stress plants were maintained at the same water status for a week (Figures 1A–D and Supplementary Figure 1). Four replicates were maintained per treatment, and all the pots were arranged in completely randomized design. Leaf samples were collected 5 days after stress level attained 50% FC for quantifying physiological traits. On the 7th day after stress reached 50% FC, soil water status was elevated to 100% FC, and plants were allowed to recover. Morphophysiological parameters such as total leaf area (TLA) and biomass were measured a week after recovery.

### Relative Water Content (%)

Relative water content (RWC) at different stress levels were measured according to the method described by Tambussi et al. (2005). Fully expanded fresh leaves of equal size were weighed to record the fresh weight (FW). The leaves were immersed in deionized water and incubated for 5 h, and later, the saturation weight (SW) of the leaves was measured by removing the surface water gently with a tissue paper. The samples were finally oven dried at 80°C to a constant dry weight (DW). RWC was calculated as:

$$\text{RWC }(\%)\text{ =}\frac{\left(\text{FW-DW}\right)}{\left(\text{SW-DW}\right)}\times \text{100}$$

### Total Chlorophyll Estimation

Chlorophyll from the weighed leaf (0.1 g) was extracted by placing it in a known volume of acetone and dimethyl sulfoxide (DMSO) (1:1) solution until the tissue was fully decolorized. The absorbance of the solution was taken using a spectrophotometer at 645 nm and 663 nm with acetone/DMSO (1:1) as the blank (Arnon, 1949). Chlorophyll content was calculated using the following formula and expressed as mg g^−1^ fresh weight.

$$\begin{array}{l} \text{Chl a = }\frac{\left(\text{12}.\text{7 X A663}\right)-\left(\text{2}.\text{54 X A645}\right)}{\text{W }\times \text{ 1000}}\times \text{ V}\\ \text{ Chl b =}\frac{\left(\text{22}.\text{9 X A645})\;(\text{4}.\text{68 X A663}\right)}{\text{W}\times \text{ 1000}}\times \text{V}\\ \text{ Total chlorophyll = Chl a + Chl b} \end{array}$$

where W is the weight of the leaf sample, V is the volume made, and A663 and A645 represents the absorbance measured at 663 and 645 nm.

### Thermal Imaging

The leaf temperature was measured in control, gradual, and rapid stressed plants using the VarioCAM® high-resolution imaging system, long-wave infrared camera (spectral range of 7.5–14 μm), having a spatial resolution of 640 × 480 pixels, accuracy of ± 1.5 K (0–100°C) or ±2 of reading and temperature range from −40 to +1,200°C. All the measurements were made from the same distance. Images were analyzed using thermographic software IRBIS® remote 3.0^*^ (Jenoptic, Germany). The leaf areas of interest from each image were outlined using imager's software for estimating average leaf temperature.

### Quantification of Superoxide, Hydrogen Peroxide, and Hydroxyl Radicals

Quantification of superoxide (${\text{O}}_{2}^{-}$) and hydrogen Peroxide (H_2_O_2_) was carried out using nitro blue tetrazolium chloride (NBT) and *diaminobenzedine* (DAB) staining assays, respectively, as described by Kumar et al. (2014). Deoxyribose assay (Aruoma, 1994) was adopted to quantify hydroxyl radicals (^•^OH). The concentration of these free radical was expressed as absorbance × 1,000.

### Schiff Base Method for Quantifying Lipid Peroxidation

Aldehyde produced as result of lipid peroxidation was visualized using the Schiff base assay (Aswathi et al., 2018). Excised leaf tissues were immediate immersed into the test tube containing 5 ml of 10% (v/v) Schiff's reagent solution. The tissue samples dipped in Schiff's reagent were vacuum infiltrated for an hour and then rinsed with distilled water to remove the stain adhered to the tissue surface. For the proper visualization of the staining, the chlorophyll was removed using the bleaching solution [acetic acid/glycerol/ethanol (1:1:3, v/v/v) solution]. Further, samples were carefully mounted on the slide, and photographs were taken under a microscope at 10 × magnification.

### Extraction and Quantification of Malondialdehyde, Methylglyoxal, and Amadori Product

Plant samples (0.1 g) obtained from each of the stress and control replicates were immersed in liquid nitrogen. The samples were homogenized with 5% (w/v) trichloro acetic (TCA) and centrifuged at 12,000 rpm for 15 min at 4°C. The supernatant was used to determine malondialdehyde (MDA) concentrations using an ultraviolet–visible spectrophotometer using Nisarga et al. (2017) method. The concentration of methylglyoxal (MG) was calculated by homogenizing the plant sample into a known amount of distilled water in accordance with Nareshkumar et al. (2020). The Amadori products were measured according to the methods described by Nisarga et al. (2017).

### Quantification of Membrane Damage by the Evans Blue Technique

Leaf samples collected from control and stress were immersed in 0.25 g Evans blue solution prepared in CaCl_2_ (0.1 M, pH 5.6) and incubated for 1 h. To remove the unbound dye on the surface, the samples were rinsed thoroughly with water. Further, the samples were treated with 1% sodium dodecyl sulfate (SDS) to release the bound dye from the cell, and absorbance was recorded at 600 nm (Vijayaraghavareddy et al., 2017).

### Quantification of Total Antioxidant Capacity

The total antioxidant capacity of the samples collected during the stress was quantified using the phosphomolybdenum method (Prieto et al., 1999). The fresh leaf sample (0.5 mg ml^−1^) extracted in 50% ethanol was mixed with the reagent solution containing 0.6 M H_2_SO_4_, 28 mM sodium phosphate, and 4 mM ammonium molybdate in 1:3 ratios. After incubating at 95°C for 150 min, the mixture was cooled immediately to room temperature. The reagent solution (4 ml), without the sample extract, incubated at the same conditions, was used as blank. Total antioxidant capacity (TAC) was measured spectrometrically at A695 nm, and values were expressed as equivalents of tannic acid (TAE).

### Quantification of Proline Content

Proline osmotic stress marker was estimated based on the protocol by Bates (1973). Leaf tissues (0.1 g) of both stressed and non-stressed seedlings were separately homogenized with 5 ml of 3% sulfosalicylic acid. Supernatants were collected by centrifugation at 12,000 rpm for 15 min for the estimation. To the 2 mL of supernatant, equal amounts of acid ninhydrin and glacial acetic acid were added. After boiling the mixture at 100°C for 1 h, samples were frozen in ice to terminate the reaction. Toluene (4 ml) was added to this mixture and vortexed vigorously for a few seconds. The mixture was then allowed to settle, and absorbance of the colored upper layer was read at 520 nm. The proline content was calculated separately with the help of a standard curve and expressed as μg g^−1^ fresh weight.

### Morphophysiological Parameters

Total leaf area (TLA) was measured by specific leaf area (SLA) method. SLA was calculated by measuring leaf area of three representative leaves from the plants as described by Reddy et al. (2020). TLA was calculated by multiplying total leaf weight with SLA. Total biomass was measured a week after recovery. Stem, leaves, and roots were separated and oven dried at 80°C for 3 days. Total leaf, stem, and root weight was computed to arrive at total biomass.

### RNA Extraction

To assess the expression levels of genes involved in scavenging mechanism, the total RNA was extracted from the leaves harvested from control, gradual, and rapidly stressed seedlings using the phenol–chloroform method (Datta et al., 1989). The quality and quantity of RNA was confirmed at A260/280 and A260/230 wavelength ratios using the NanoDrop® ND-1000 spectrophotometer. Complementary DNA (cDNA) was synthesized from the total RNA using oligo (dT) primers by M-MLV reverse transcriptase mediated reverse transcription. Quantitative RT-PCR reaction was carried out using iQ5 real-time PCR detection system (Bio-Rad) with a final volume of 20 μl using the TAKARA SYBR Green qPCR Kit. The PCR program was conducted with the following cycling conditions: 94°C for 3 min followed by a 25-cycle program (94°C, 30 s; 52–58°C, 30 s; 72°C, 40 s, and 72°C, 5 min per cycle). Ubiquitin was used as the endogenous reference gene to normalize the data. The relative expression of the scavenging genes (primer details are given in Supplementary Table 1) was estimated using 2^−Δ*ΔCT*^ method (Livak and Schmittgen, 2001; Yoo et al., 2017; Çelik et al., 2019; Rauf et al., 2019).

## Experiment 2

Gradual moisture stress was imposed to 17 selected germplasm lines with similar phenology at flowering (85DAS) (Supplementary Table 2) during the Kharif season of 2019 to confirm the trait diversity, particularly for acquired tolerance traits. Phenotyping for acquired tolerance traits was carried out by raising the plants in mini-lysimeter drought-simulator platform. Experiment was carried out in 20-L capacity containers filled with red sandy loam soil and farm yard manure in 3:1 ratio. Three replicates each for control (100% FC) and gradual stress treatment were maintained per genotype, and all the pots were arranged in completely randomized design. A similar induction protocol, as described above, was followed with 5% reduction in soil moisture status per day. The dry down protocol was initiated at 5 days before flowering (80DAS) (during the booting stage). Plants reached 50% FC on the 10th day after stress imposition (90DAS). Plants were maintained at 50% FC for 1 week, and later, the stressed containers were brought back to 100% FC and continued till maturity. However, a set of plants were also raised in similar containers and maintained at 100% FC, as control. During stress, leaf samples from both stressed and control plants were collected and the ROS (superoxide, hydrogen peroxide), MDA content, total antioxidants, and proline content were quantified using the protocol described above. At harvest, shoot, leaves, and roots were separated and oven dried at 65°C for 3 days, and weighed values were computed to obtain the total biomass. The total numbers of filled grains and chaffy grains were counted manually to arrive at spikelet fertility.

$$\text{Spikelet fertility }\left(\%\right)\text{=}\frac{\text{number of filled grains}}{\text{total number of grains}}\;\times \text{100}$$

### Statistical Analysis

All data were analyzed to check for statistical significance using the software GENSTAT Ver. 12.1(http://www.genstat.co.uk/). To reveal differences between cultivars and between treatments, a two-way analysis of variance was carried out. Pearson's correlation coefficients were determined using r corr package in R version 4.0.3 (https://www.r-project.org/).

## Result

### Differences in Stress Response in Two Contrasting Rice Genotypes

**Figure 2:**
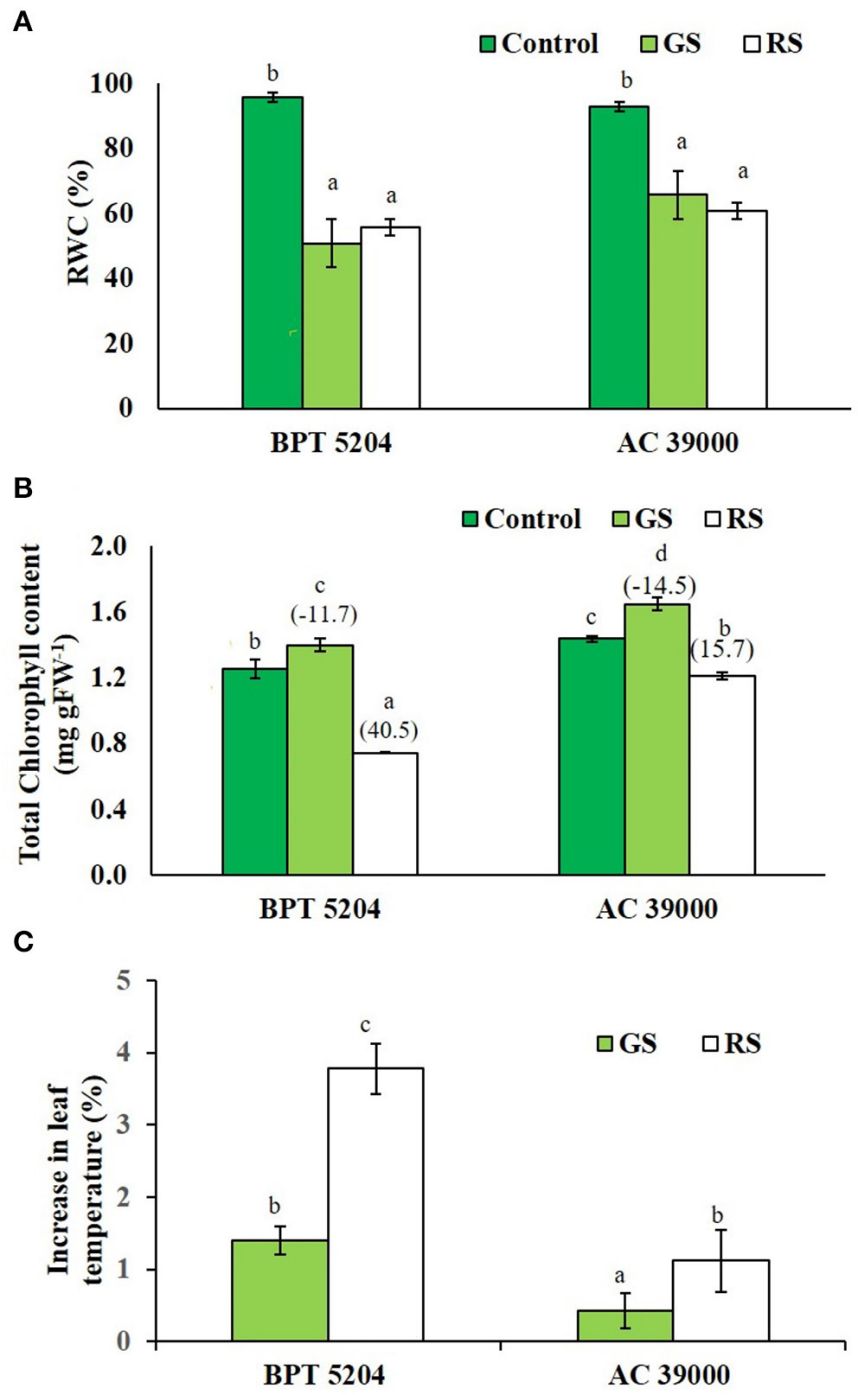
Effect of moisture stress on **(A)** relative water content (RWC), **(B)** total chlorophyll, and **(C)** percent reduction in leaf temperature over control in two rice genotypes, BPT 5204 and AC 39000, in control, gradual stress (GS), and rapid stress (RS) conditions. Values are mean of three replicates. Values in parenthesis indicates percent reduction over control for mean. Different letters indicate significant different between genotypes and treatment at *P* < 0.05.

The leaf relative water content (RWC) of both the contrasting genotypes, BPT 5204 and AC 39000, decreased significantly under stress (Figure 2A). Interestingly, the chlorophyll content marginally increased in both the genotypes under gradual stress treatment, while it significantly decreased in rapid stress treatment (Figure 2B). The moisture stress tolerant AC 39000 showed only a 15.7% reduction in total chlorophyll, while the corresponding reduction was 40.5% in BPT 5204 under RS compared to their respective controls (Figure 2B).

Leaf temperature increased significantly under stress in both the genotypes (Figure 2C), and the extent of increase in leaf temperature was less when the stress progressed gradually. Between the genotypes, the stress tolerant genotype, AC 39000, maintained a significantly cooler canopy, compared with BPT 5204.

### Gradual Stress Primes the Plant for Better Management of ROS and RCCs Under Severe Stress

**Figure 3:**
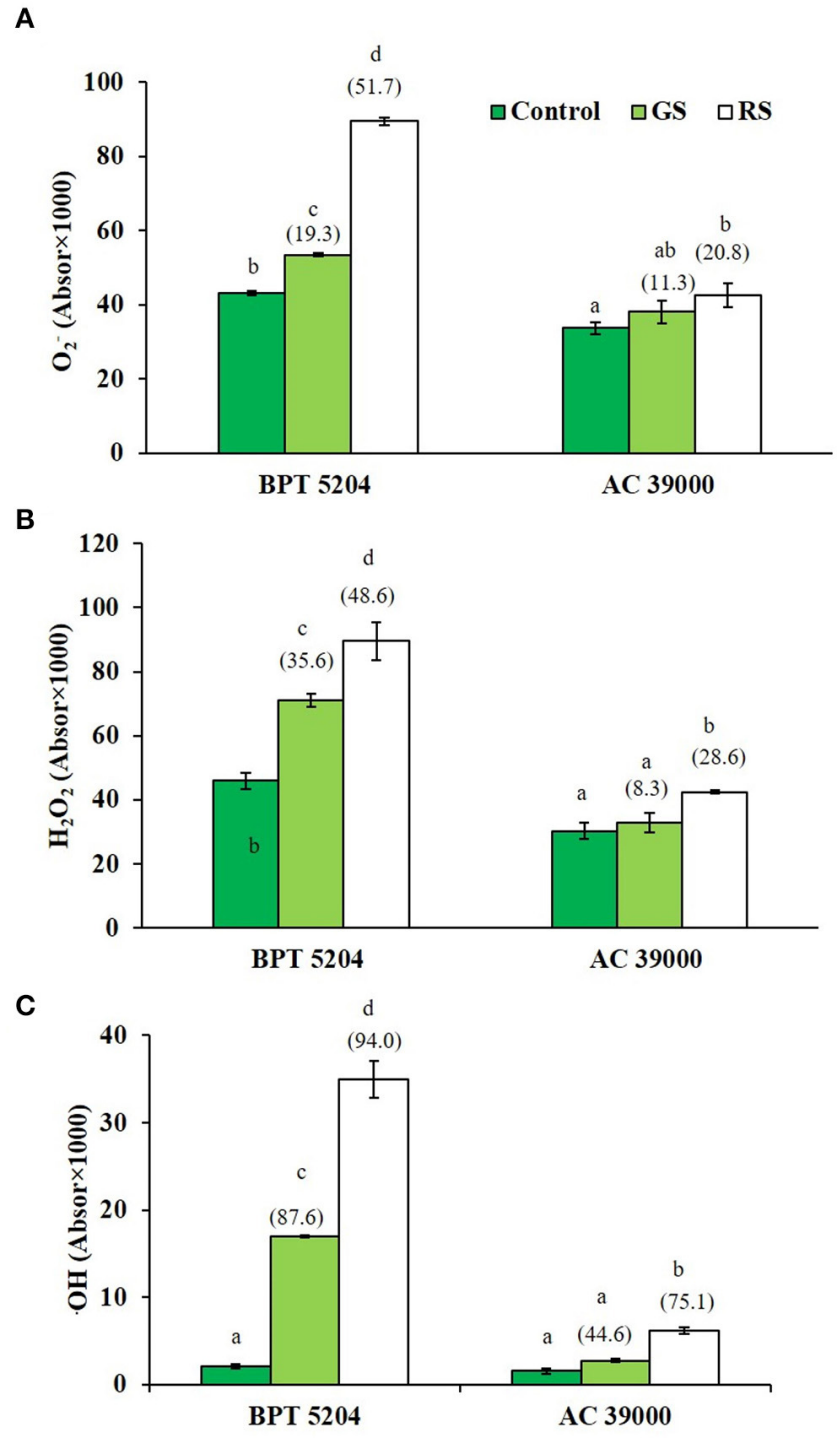
Effect of moisture stress on reactive oxygen species production. **(A)** ${\text{O}}_{2}^{-}$ using nitro blue tetrazolium chloride (NBT) staining, **(B)** H_2_O_2_ using diaminobenzedine (DAB) staining, and **(C)** ^•^OH radical, in two rice genotypes, BPT 5204 and AC 39000, in control, gradual stress (GS), and rapid stress (RS) conditions. Values are mean of three replicates. Values in parenthesis indicates percent reduction over control for mean value. Different letters indicate significant different between genotypes and treatment at *P* < 0.05.

**Figure 4:**
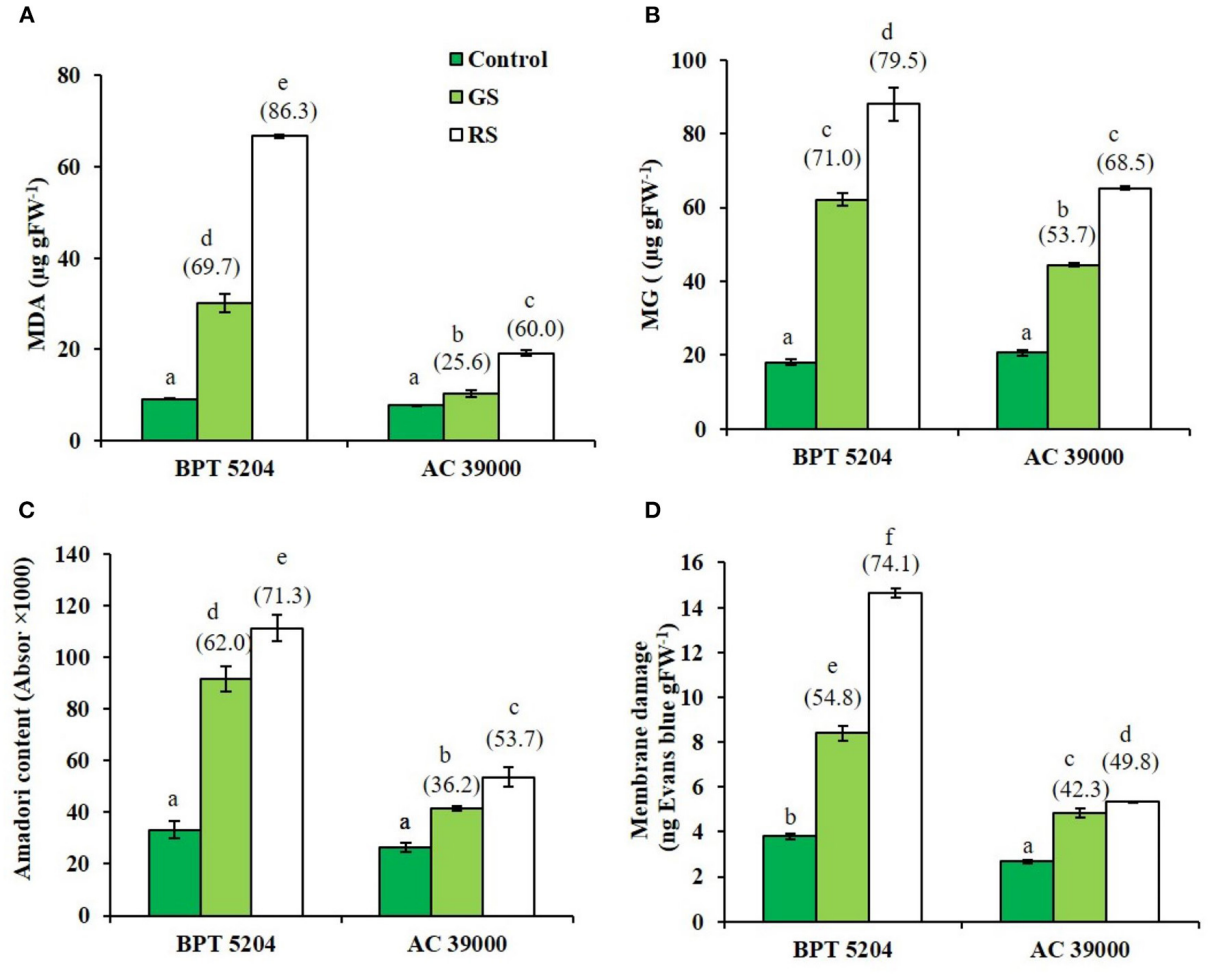
Effect of moisture stress on production of reactive carbonyls and membrane damage. Measurements were made in control, gradual moisture stress (GS), and rapid moisture stress (RS) conditions at seedling stage. **(A)** Malondialdehyde (MDA), **(B)** methyl glyoxol (MG), **(C)** Amadori content, and **(D)** membrane damage using Evans blue staining. Values are mean of three replicates. Values in parenthesis indicate percent reduction over control for mean. Different letters indicate significant different between genotypes and treatment at *P* < 0.05.

Levels of ROS such as H_2_O_2_, ${\text{O}}_{2}^{-}$, and ^•^OH radicals were significantly increased when plants were exposed to moisture stress. Increase in ROS levels was remarkably less when plants were gradually stressed (Figures 3A–C), indicating a better management of ROS levels when stress progressed gradually. The images of the staining procedure for quantification of ROS are given in Supplementary Figure 2. All ROS were significantly higher in the moisture stress-sensitive BPT 5204 compared with AC 39000. Similarly, the RCCs such as MDA, MG, and Amadori compounds were higher in BPT 5204 under both gradual and rapid stresses compared with AC 39000 (Figures 4A–C). MG increased by 71 and 79% under gradual and rapid stress conditions, respectively, over control in the stress-sensitive BPT 5204 (Figure 4B), whereas MG increased by 53 and 68%, respectively, in gradual and rapid stress in AC 39000. Amadori compounds also showed a typically similar trend both between genotypes and between gradual and rapid stress progression representing a significant difference in RCC levels across genotypes as well as stress progression (Figure 4C).

**Figure 5:**
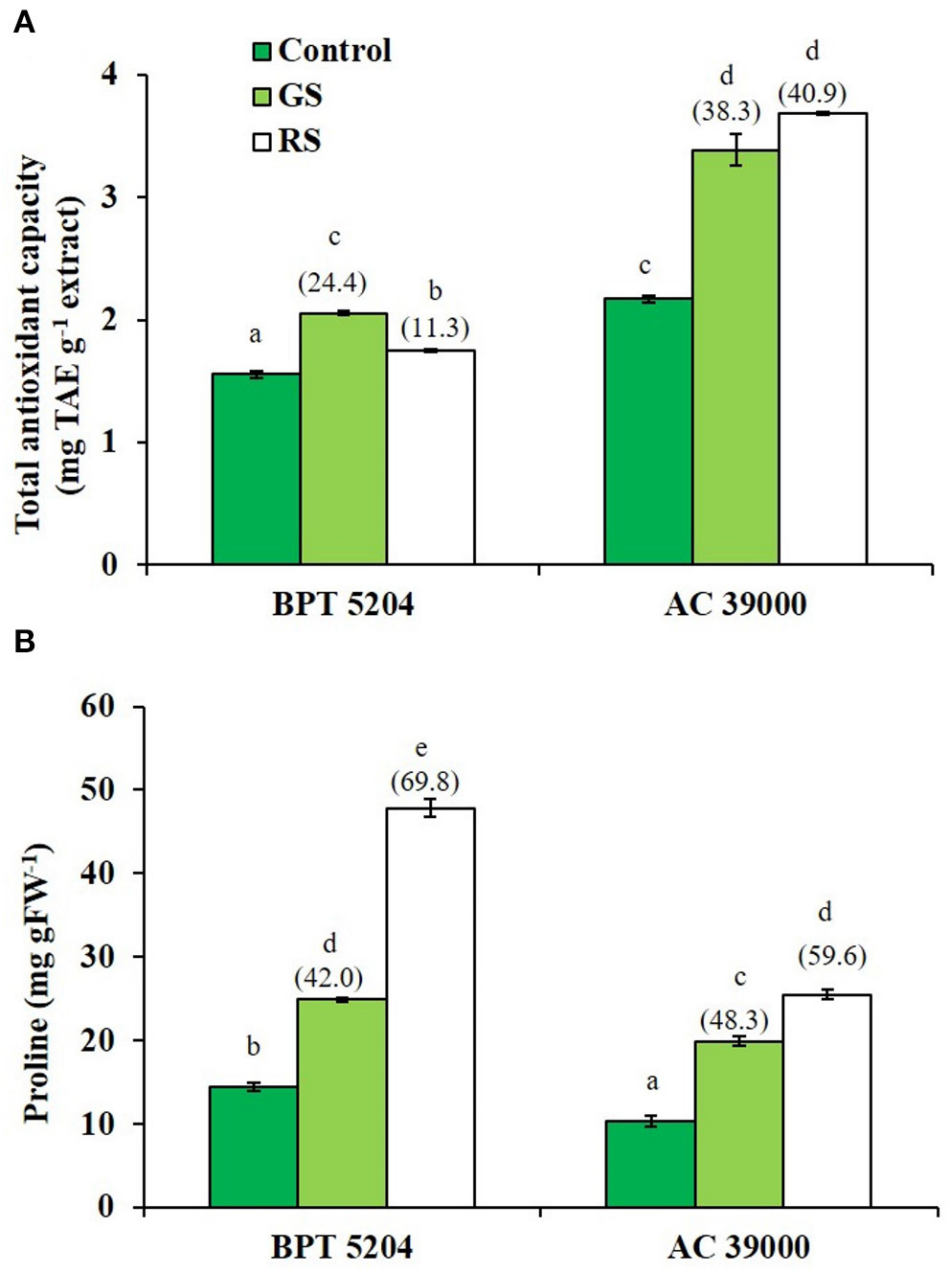
Enhancement in the scavenging activity accompanying with gradual stress (GS) and rapid stress (RS) in terms of **(A)** total antioxidant content (TAC) calculated as tannic acid equivalence (TAE) and **(B)** proline content in two rice genotypes, BPT 5204 and AC 39000. Values are mean of three replicates. Values in parenthesis indicate percent reduction over control for mean value. Different letters indicate significant different between genotypes and treatment at *P* < 0.05.

ROS and RCCs would lead to destruction of cell membrane integrity, primarily through peroxidation of membrane lipids. The increased RCC levels were perfectly supported with the loss of cell membrane integrity as evidenced by a 54 and 74% increase in staining of Evan's Blue, respectively, under gradual and rapid stress treatments in BPT 5204 (Figure 4D). The corresponding values for loss of cell membrane integrity were 42 and 49% in AC 39000 under gradual and rapid stress progression (Figure 4D). The total antioxidant capacity also showed a similar trend between stress imposition protocol. The tolerant genotype, AC 39000, showed a much higher increase in total antioxidant capacity than BPT 5204 (Figure 5A). The moisture stress-tolerant genotype, AC 39000, accumulated noticeably lower proline content both in rapid and gradual stress treatments compared to BPT 5204 (Figure 5B).

### Gradual Stress Induces ROS/RCC Scavenging Genes Better Than the Rapid Stress

**Figure 6:**
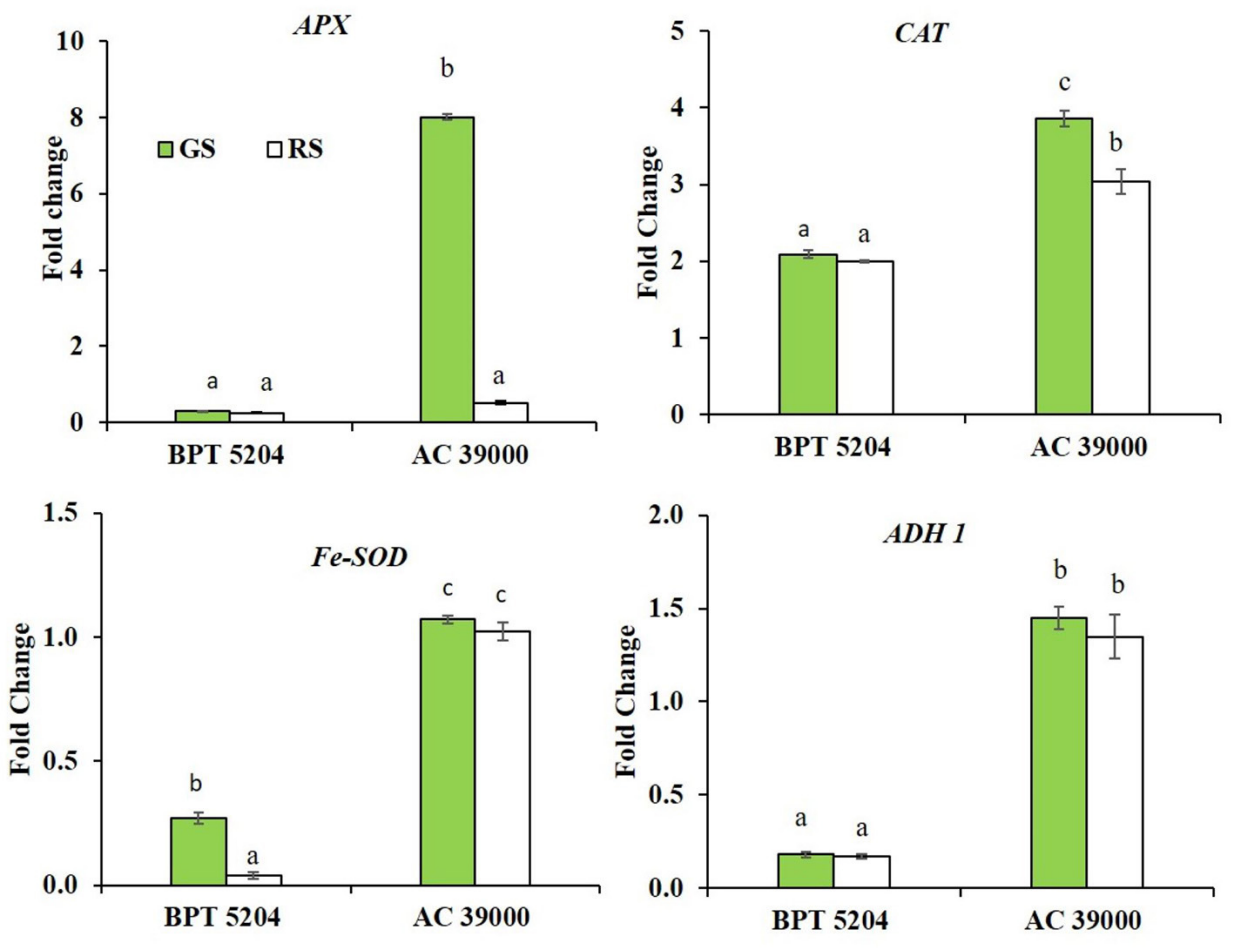
Real-time quantitative RT-PCR analysis for genes associated with free radical scavenging in two rice genotypes BPT 5204 and AC 39000 under gradual stress (GS) and rapid stress (RS) conditions. Data represent the fold change in expression levels over control. Values are mean of three replicates. Different letters indicate significant different between genotypes and treatment at *P* < 0.05.

Expression of genes involved in scavenging ROS and RCCs, viz., *ascorbate peroxidase (APX), catalase (CAT), superoxide dismutase (Fe-SOD)*, and *alcohol dehydrogenase 1 (ADH1)* showed significant genotypic and stress response. The differential expression of all the genes was significantly higher for AC 39000 compared with BPT 5204 (Figure 6). Among the gene expression examined, *APX* and *CAT* gene expressions were higher under gradual stress treatment in AC 39000. But for BPT 5204, only *Fe-SOD* gene expression responded to the rate of stress development, indicating a lack of propensity to respond to stress in the sensitive BPT 5204 (Figure 6). However, *ADH1* expression, although significantly high for AC 39000, did not respond to stress imposition protocols in either of the genotypes.

### Seedling Growth vs. Stress Imposition Protocol

**Figure 7:**
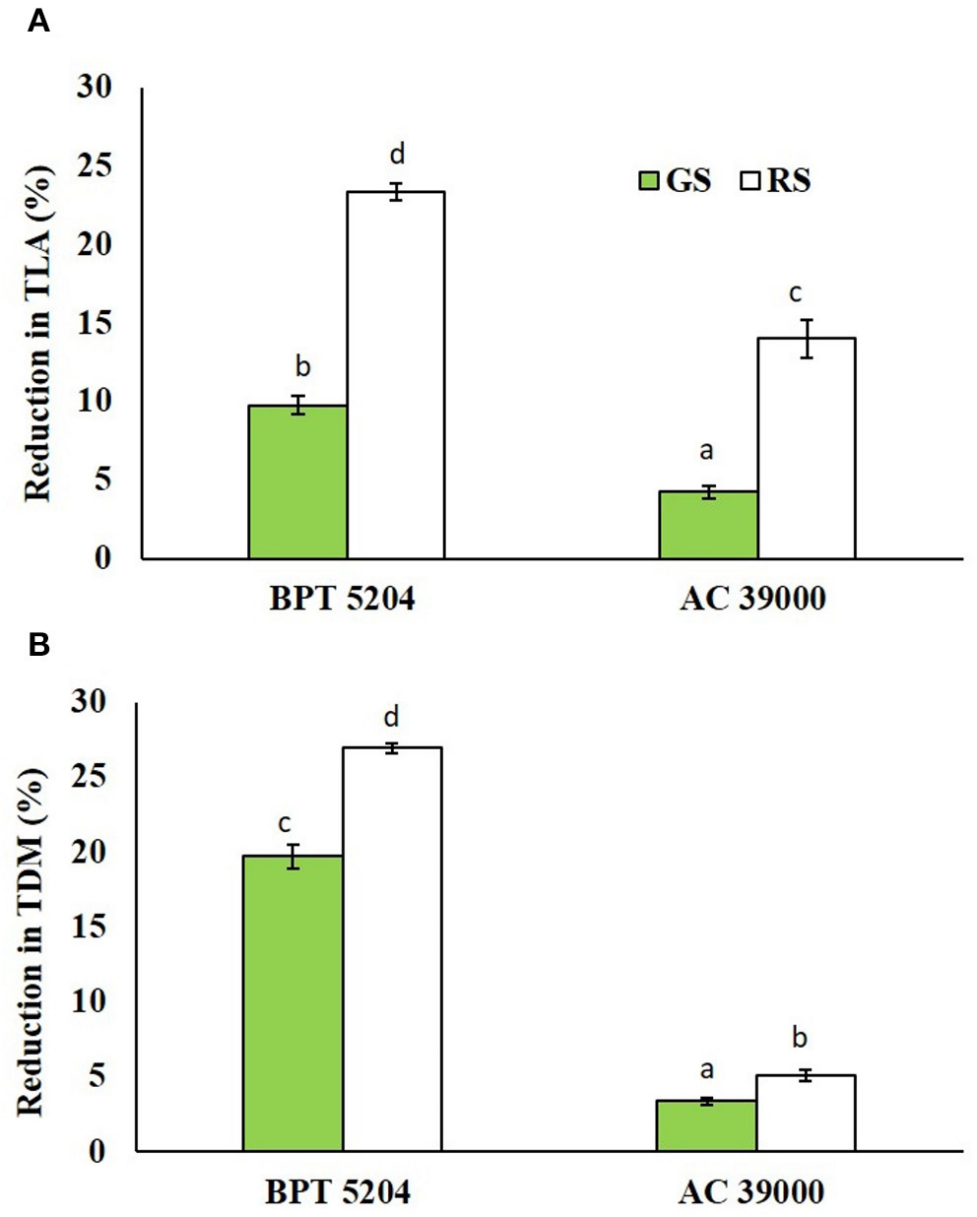
Effect of the gradual stress (GS) and rapid stress (RS) in two rice genotypes (BPT 5204 and AC 39000) on **(A)** percent reduction in total leaf area (TLA) and **(B)** total dry matter (TDM) over control. Values are mean of three replicates. Different letters indicate significant different between genotypes and treatment at *P* < 0.05.

The reductions in leaf area and biomass accumulation were significantly high under rapid stress treatment in both genotypes. The moisture stress-sensitive genotype, BPT 5204, displayed significantly higher reduction in both leaf area and biomass than the tolerance AC 39000 under both moisture stress treatments (Figures 7A,B).

### Genetic Variability in Acquired Tolerance Traits

Results from Experiment 1 clearly demonstrated that gradually progressing stress primed the upregulation of oxidative stress protective mechanisms. To examine if germplasm accessions differ in their propensity to respond, a set of 17 diverse rice germplasm accessions were screened for their response to gradual stress at the flowering phase.

The increase in ${\text{O}}_{2}^{-}$ varied between 10.8 and 69.7% under stress among genotypes, and the percent increase in H_2_O_2_ ranged between 8.06 and 62.51%, representing a significant genotypic variability (Table 1). The values of all ROS and RCCs for all genotypes is presented in Supplementary Tables 3, 4. The increase in MDA content due to stress ranged from 5 to 34%. Stress-induced membrane damage also significantly increased in all the genotype ranging from 3.9 to 56.3% (Table 1). The upregulation of scavenging activity also showed significant variability of 4.9–54.8%. Similarly, the increase in proline content showed significant genotypic variability among the genotypes (11.2–79.4%). Genotypes GEN 224 and GEN 541 revealed lowest percent increase in ROS levels, lipid peroxidation, and a lower extent of membrane damage (Supplementary Tables 3, 4). Increased levels of TAC, which is an important component of acquired tolerance, closely matched with lower levels of ROS, RCC, and membrane damage in these genotypes (Table 1 and Supplementary Tables 3, 4).

**Table 1:** Descriptive statistics for superoxide content (${\text{O}}_{2}^{-}$), hydrogen peroxide (H_2_O_2_), malondialdehyde (MDA), membrane damage, total antioxidant capacity and total proline among 17 rice germplasm lines.

**Plant trait**	**Treatment**	**Mean**	**Minimum (Percent change)**	**Maximum (Percent change)**	**SE**	**LSD_**0.05**_**	**CV (%)**
${\text{O}}_{2}^{-}$ (Absorb × 1,000)	Control (100% FC)	364.6	10.8	69.7	0.16	0.26	29.9
	Stress (50% FC)	693.4					
H_2_O_2_ (Absorb × 1,000)	Control (100% FC)	126.5	8.1	62.5	0.05	0.08	26.7
	Stress (50% FC)	233.0					
MDA (μgg^−1^ FW)	Control (100% FC)	0.6	5.0	34.0	0.06	0.10	9.0
	Stress (50% FC)	0.7					
Membrane damage (ng Evans blue g^−1^ FW)	Control (100% FC)	14.6	3.9	56.3	3.31	5.54	17.7
	Stress (50% FC)	22.4					
Total antioxidant capacity (mg TAE g^−1^ extract)	Control (100% FC)	2.5	4.9	54.8	0.35	0.57	10.7
	Stress (50% FC)	3.5					
Total proline (μgg^−1^ FW)	Control (100% FC)	8.7	11.2	79.4	2.17	3.52	13.7
	Stress (50% FC)	15.3					

*LSD _0.05_; least significant difference at 5%*.

Spikelet fertility decreased under stress ranging from 12.11% in GEN 541 to 39.39% over control in GEN 484, representing a significant genotypic variability (Table 2 and Supplementary Table 5). Similarly, percent reduction in biomass and yield per plant were also recorded, and its range in their respective reduction over control are given in Table 2 and Supplementary Table 5.

**Table 2:** Descriptive statistics for total dry matter production (TDM), spikelet fertility (SF) and yield among 17 rice germplasm lines.

**Plant trait**		**Mean**	**Minimum (percent change)**	**Maximum (percent change)**	**SE**	**LSD_**0.05**_**	**CV (%)**
TDM (gpot^−1^)	Control (100% FC)	218.5	1.0	27.5	14.2	5.4	7.1
	Stress (50% FC)	187.0					
SF (%)	Control (100% FC)	88.7	12.11	39.4	3.6	1.4	4.7
	Stress (50% FC)	65.2					
Yield (gpot^−1^)	Control (100% FC)	112.6	13.6	50	9.9	12.8	9.9
	Stress (50% FC)	76.4					

*LSD _0.05_; least significant difference at 5%*.

### Correlation Analysis for the Measured Parameters

**Figure 8:**
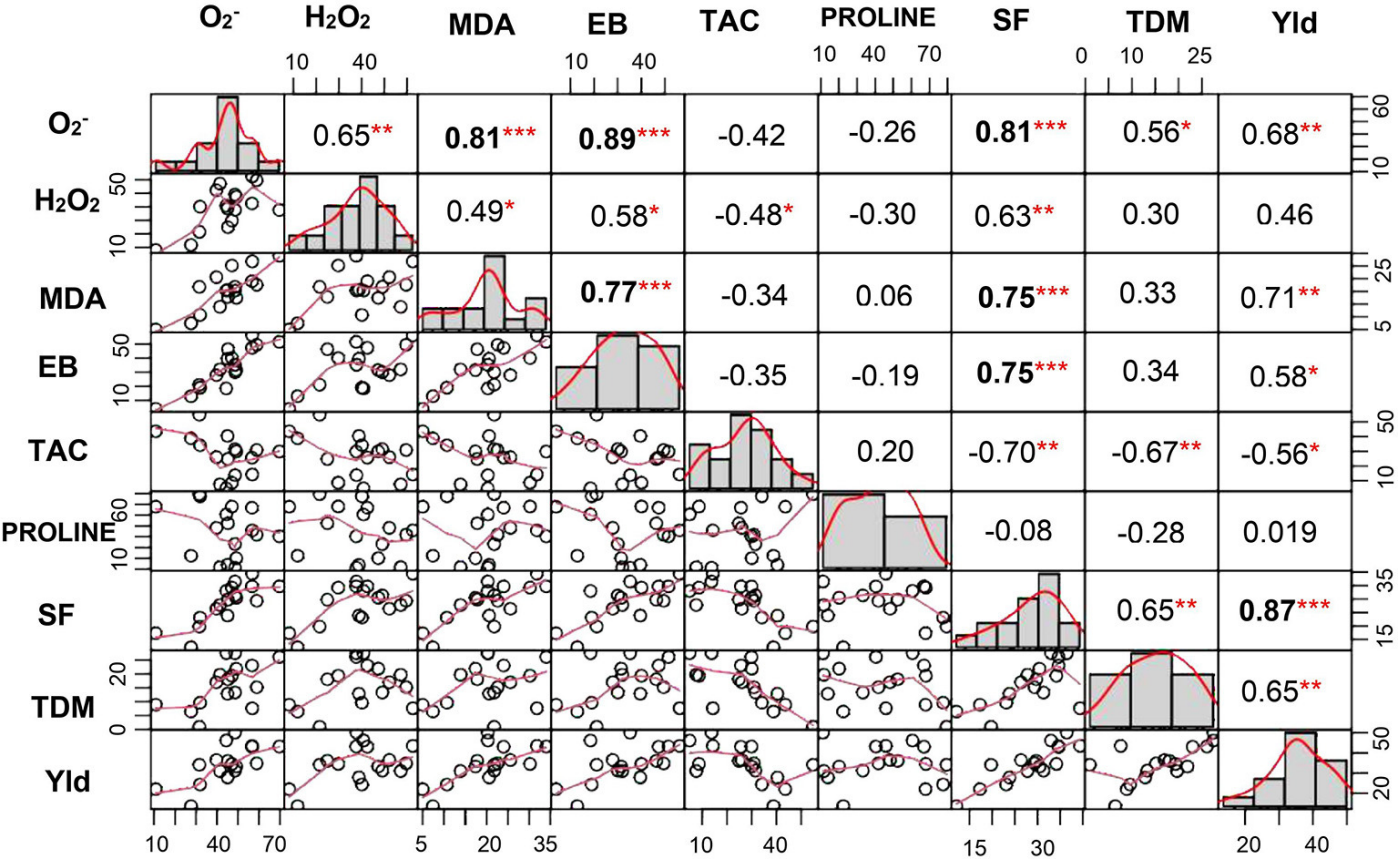
Correlation coefficient matrix of percent change in various acquired and morphophysiological traits. In the diagonal panel, frequency of phenotypic traits is given as histograms. The lower panel is a scatter plot with a red line indicating the best fit. The upper panel represents the value of the Pearson correlation coefficient. *, **, and *** indicate significant correlations at *P* < 0.05, *P* < 0.01, and *P* < 0.001, respectively. ${\text{O}}_{2}^{-}$, superoxide content; H_2_O_2_, hydrogen peroxide content; EB, Evans blue; MDA, malondialdehyde; TAC, total antioxidant capacity; Yld, yield; SF, spikelet fertility; TDM, total dry matter among 17 lines of rice germplasm. All parameters were assessed at reproductive stage and correlation matrix was done for percent change over control.

A significant correlation was observed between ${\text{O}}_{2}^{-}$ and H_2_O_2_ at *P* < 0.01. Similarly, lipid peroxidation end product also showed strong correlation with ${\text{O}}_{2}^{-}$ and H_2_O_2_ (at *P* < 0.001 and *P* < 0.05, respectively). Membrane damage measured by Evans blue technique also showed significant positive correlation with ${\text{O}}_{2}^{-}$ (*P* < 0.001), H_2_O_2_ (*P* < 0.05), and MDA (*P* < 0.001). TAC showed negative association with both the ROS and was significant only with H_2_O_2_ (*P* < 0.05). Similarly, spikelet fertility (*P* < 0.01), TDM (*P* < 0.01), and yield (*P* < 0.05) were also negatively correlated with TAC, whereas spikelet fertility and yield showed positive correlation with ROS, MDA, and membrane damage (Figure 8). Spikelet fertility was also strongly related to percent reduction in yield (*P* < 0.001) under stress (Figure 8), which explained around 75% of variability in yield loss (Supplementary Figure 3). These results emphatically illustrate the role of acquired tolerance traits associated with reduction in generation and/or improved scavenging of ROS, in regulating rice grain yield. Reduced ROS and RCC levels maintain grain yield through their influence on spikelet fertility. The two most stress tolerant genotypes GEN 541 and GEN 224 maintained significantly higher spikelet fertility and yield under stress compared with the two most sensitive genotypes, viz., GEN 484 and GEN 443. These two groups of genotypes significantly differed in their ROS and RCC levels (Table 3).

**Table 3:** Comparison of total yield and acquired tolerance traits in genotypes with maximum and minimum reduction in spikelet fertility (SF).

	**Genotype**	**Yield (stress) (gpot^**−1**^)**	**${\text{O}}_{2}^{-}$ (%)**	**H_**2**_O_**2**_ (%)**	**Membrane damage (%)**	**MDA (%)**	**TAC (%)**
Lowest reduction in SF	GEN 541	106.8 ± 3.2	27.6 ± 1.9	11.8 ± 0.5	12.9 ± 0.7	7.4 ± 0.6	38.8 ± 2.8
	GEN 224	105.7 ± 2.0	10.8 ± 1.7	8.1 ± 0.6	3.9 ± 0.2	5.0 ± 0.7	43.6 ± 0.7
Highest reduction in SF	GEN 484	59.7 ± 4.1	56.8 ± 0.9	62.5 ± 1.0	56.3 ± 1.3	31.8 ± 3.4	14.3 ± 0.5
	GEN 443	43.1 ± 1.0	48.6 ± 0.1	37.7 ± 0.0	35.3 ± 2.6	20.3 ± 3.5	13.8 ± 0.9

*Percent increase in ROS and RCC (${\text{O}}_{2}^{-}$,H_2_O_2_), membrane damage, malondialdehyde (MDA) and total antioxidant capacity (TAC) among selected four rice germplasm lines. (Note: Refer Supplementary Table 1 for the names of genotypes)*.

## Discussion

Plants cope with stresses through development of several adaptive strategies that can be generally classified as constitutive and acquired traits based on the pattern of their expression (Xin, 1998; Collins et al., 2008; Sheshshayee et al., 2018). While constitutive traits are time integrated, acquired tolerance traits/mechanisms are significantly upregulated only when plants experience milder levels of stress. Most of the abiotic stresses like drought progress gradually and prime plant responses by upregulating specific cellular mechanisms that provide decisive survival advantage during severe or even lethal levels of stress (Mittler, 2006; Galeano et al., 2019; Kollist et al., 2019; Zhang et al., 2020). Based on this premise, a simple temperature induction response technique was developed (Uma et al., 1995; Senthil-kumar et al., 2003; Vidya et al., 2017), which provided the initial screening option. The findings from these experiments on acquired ability to manage ROS and RCCs were done with young, germinated seedlings (heterotrophic stage). The skepticism about the validation of this approach in screening for acquired tolerance during more advanced phenological stages has been one of the reasons for the lack of keenness in adopting acquired tolerance traits in crop improvement.

Screening for variability in induction response to drought stress in plants at advanced developmental stages, especially grand growth, flowering and anthesis, and grain filling periods, has been a challenge as methodology for imposing drought stress in a precisely controlled progression was not possible (Poorter et al., 2012; Manmathan and Lapitan, 2013). In container experiments, at phenological phases when canopy cover is maximum, withholding irrigation may lead to a rapid development of stress depending on soil texture and prevailing weather conditions. On the contrary, field-grown plants experience a gradual progression of soil moisture stress, which provides an opportunity for inducing acquired tolerance. However, differences in water mining abilities and transpiration rates between accessions creates variability in the tissue water relations and hence in acquired tolerance levels. Hence, research leads from rapid stress development in container experiments or field drought studies could not be translated for crop improvement, except for a few transcription activators (Snow and Tingey, 1985; Mishra et al., 2012; Marchand et al., 2013).

The transpiration-interfaced automated drought-simulator phenomics facility established at the Department of Crop Physiology, University of Agricultural Sciences, Bengaluru, India, significantly circumvents this lacuna (Vijayaraghavareddy et al., 2020a). The programmable “dry-down” protocol available in this facility is capable of developing a user-defined stress progression in containers, mimicking the field conditions and hence provide an excellent option to screen for plant response to gradually progressing stress (Figures 1A–D and Supplementary Figure 1).

In this study, we initially compared the plant response to rapid and gradual stress progression treatments using a drought tolerant and a sensitive cultivar of rice and analyzed the differences in acquired tolerance mechanisms. One of the most prominent *de novo* mechanisms of developing tolerance to stress is the management of oxidative burst. Production of ROS appears like an inevitable consequence when plants experience stress (Cruz de Carvalho, 2008). However, plants have also developed several mechanisms of scavenging these ROS and RCCs to manage the oxidative stress (Rogers and Munné-Bosch, 2016). Several of the known oxidative management strategies are induced only under stress and hence represent an important stress coping strategy. We examined the ROS and RCC levels under induced and uninduced seedlings and standardized the evaluation methodology. Subsequently, a set of diverse germplasm accessions was screened for oxidative response as a reflection of acquired tolerance under gradually progressing stress condition at flowering stage of the crop. We hypothesized that the induction of acquired tolerance in leaves would maintain the source activity and hence help in the spikelet growth and seed development.

### Coping With Moisture Stress: Rapid vs. Gradual Stress

The first experiment revealed that the plants acquired tolerance when the stress progression was gradual, and the propensity to respond though was seen in both the genotypes; it was significantly higher in the tolerant AC 39000.

Leaf tissue water status determines the expression of mechanisms associated with acquired tolerance (Jones, 2007). Water mining associated with root traits generally determine the tissue water status. Hence, under field conditions, a deep-rooted plant often do not show water-deficient symptom even when the soil moisture content decreases (Yadav and Sharma, 2016). In the phenomics facility, the transpiration-interfaced automated irrigation system brings the soil moisture status to the same level irrespective of the differences in water mining and transpiration. This facility is hence quite suitable for capturing the genetic variability in acquired tolerances. The relative water content of the sensitive and tolerant genotype was comparable both at control and water-limited treatments. A significant difference in leaf temperature between the two genotypes was noticed with AC 39000 maintaining a much cooler canopy. This genotype apparently transpired more water during the same duration than IR64 both under control and water-limited conditions (data not shown). The thermal imaging system (VarioCAM®) used to measure the leaf temperature in this study was done by specifically selecting the leaf. This spot measurement was quite precise and did not require extensive background calibration for temperature measurements.

The automated irrigation combined with a predefined dry-down protocol of the drought-simulator phenomics facility facilitated the maintenance of comparable leaf tissue water relations between genotypes as well as between stress progression treatments irrespective of differences in total leaf area between the genotypes. Hence, the differential performance of the genotypes to stress levels would be almost entirely because of the ability to upregulate acquired traits under stress (Figures 3–7). The higher levels of TAC along with the levels of expression of free radical scavenging enzymes clearly illustrated that the drought tolerant AC 39000 displayed higher abilities to upregulate free radical management mechanisms and hence “acquired” higher levels of tolerance to drought stress (Figures 5A, 6).

### Genetic Variability in Acquired Tolerance Traits and Spikelet Fertility Under Gradually Imposed Moisture Stress

The relevance of acquired tolerance in plant stress adaptation has been well-accepted (Kumar et al., 1999; Sheshshayee et al., 2018). Yet, most of the investigations attempted the assessment of these acquired tolerance traits using young, germinated seedlings. Although some experiments provided evidences that the variability in acquired tolerance measured at the heterotrophic stage of germinated seedlings can reflect stress adaptation at an advanced autotrophic stage of the crop (Raju et al., 2014), such reports are not many. The mechanism(s) of stress adaptation could be highly variable between specific phenological phases of crop growth (Vijayaraghavareddy et al., 2020b). They reported that stress occurring at flowering stage can cause maximum damage to yield due to increased spikelet sterility. We hypothesized that genotypes with superior propensity to respond to milder stress would protect the spikelets from becoming sterile. This hypothesis was tested with a set of 17 diverse germplasm accessions of rice. A gradual stress protocol was adopted to coincide with the flowering phase of each genotype.

The yield of all the 17 germplasm lines showed a significant reduction under stress (Table 2 and Supplementary Table 5). Stress during the flowering phase could have strong influence on anther dehiscence, pollen germination, stigmatic receptivity, extent of fertilization (Hedhly et al., 2003; Distefano et al., 2018; Matsui and Hasegawa, 2019), etc., leading to spikelet sterility. A strong association was also noticed between all the ROS and RCC levels with percent reduction in spikelet fertility (Figure 8). The TAC clearly revealed that a genotype that maintained ROS homeostasis through avoiding production and/or scavenging the ROS and RCCs would maintain higher grain yields under stress. The significant differential expressions of genes associated with ROS and RCC scavenging mechanisms indicate that phenotypic expression is strongly induced when plants experience mild stress and hence can be classified as acquired traits. Significant genotypic variability among germplasm accessions increases the possibility of using the best of genotypes as trait donor lines for improving acquired tolerance traits in crop improvement programs. Genotypes like GEN 224 and GEN 541 that maintained lower levels of ROS and RCCs and hence cell membrane integrity and lowest percent reduction in spikelet fertility resulted in lower reduction in yield under stress (Table 3 and Supplementary Tables 3–5).

## Conclusions

This study demonstrated, for the first time, the relevance of precise stress imposition in capturing acquired tolerance traits. Significant upregulation of these traits were noticed only when the plants are gradually stressed. The mini-lysimeter (MLM)-based drought-simulator phenomic facility provided an excellent unique option to assess the drought stress response at a specific phenological phase of crop. The study revealed that the total antioxidant capacity was strongly related to maintenance of spikelet fertility, which led to a relatively higher grain yield. The genetic variability revealed in this study increases the optimism about breeding for enhanced acquired tolerance in rice to sustain yields, especially under water saving agronomic practices like semi-irrigated aerobic cultivation.

## Data Availability Statement

The original contributions presented in the study are included in the article/Supplementary Material, further inquiries can be directed to the corresponding author/s.

## Author Contributions

VL conducted all experiments, designed experimental methodology, analyzed the data, and developed the first draft of manuscript. PV designed the experimental methodology, conducted experiments, and edited the first draft. AN helped conduct all experiments in the drought-simulator phenomics platform. VSR provided useful inputs in developing methodology and edited the first draft. VR helped in conduct of phenomics experiments and edited the first draft. UM provided very useful discussions during the formation of this program and helped in the interpretation of data and discussions and edited the first draft. SS conceived the idea, directed the research, provided overall supervision, and provided funds for this investigation. All authors contributed to the article and approved the submitted version.

## Conflict of Interest

The authors declare that the research was conducted in the absence of any commercial or financial relationships that could be construed as a potential conflict of interest.
