# Supplementary material for: Induction of Acquired Tolerance Through Gradual Progression of Drought Is the Key for Maintenance of Spikelet Fertility and Yield in Rice Under Semi-irrigated Aerobic Conditions
Source: Front Plant Sci. 2021 Feb 18;11:632919. doi: 10.3389/fpls.2020.632919 (PMC7930615; doi:10.3389/fpls.2020.632919)
Supplement: Supplementary file 1 [file Data_Sheet_1.pdf]

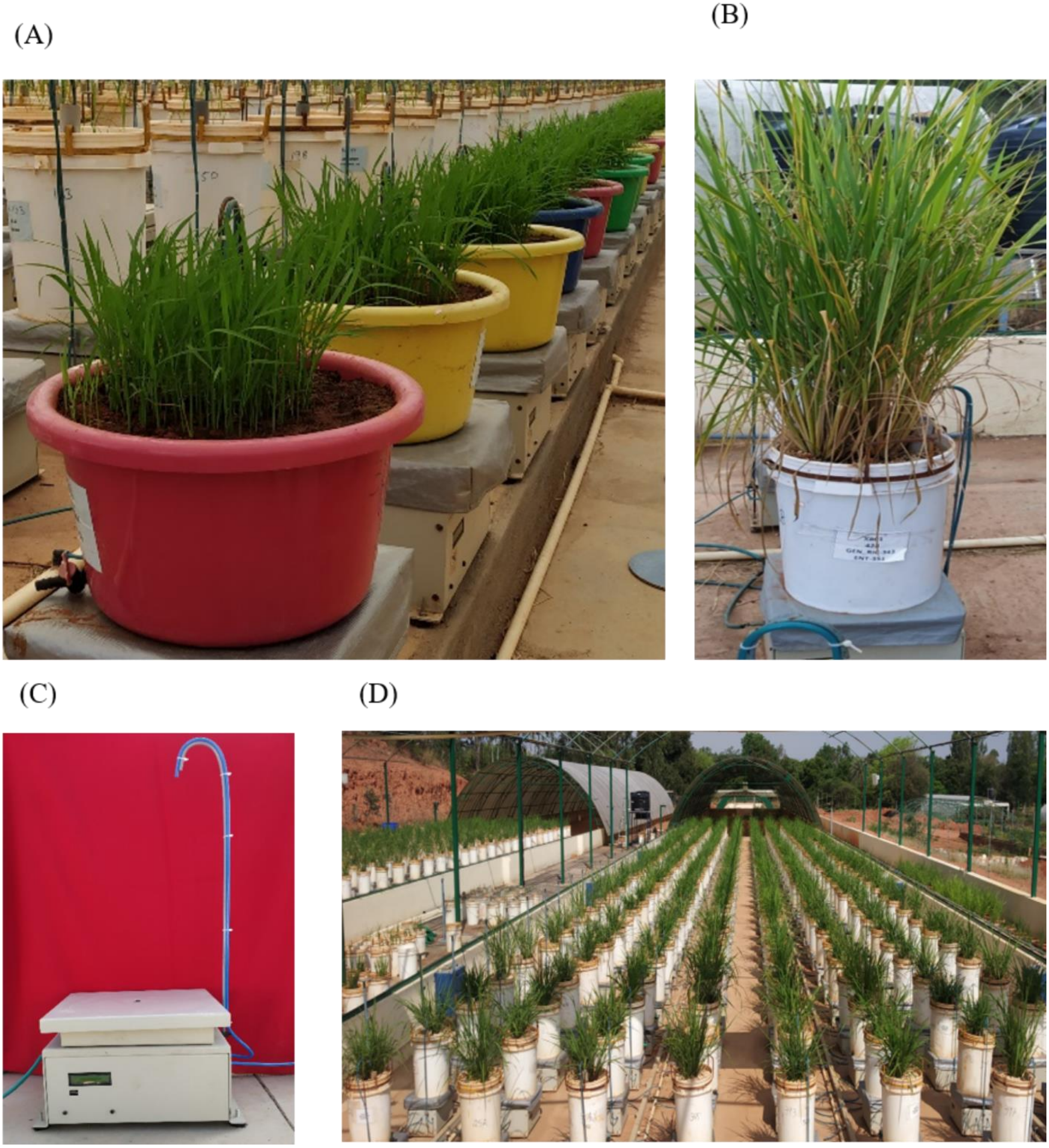

Figure S1: Minilysimeter (MLM) based drought-simulator platform for stress imposition protocol in rice. (A) Experiment 1 with 10 L capacity pots for protocol standardization during the seedling stage and (B) pots of 20 L capacity for inducing gradual stress in 17 genotypes were placed on lysimeters with the automated irrigation facility. (C) Load cell balance platform along with an irrigation pipe. (D) Overall view of the drought-simulator phenomics facility at University of Agricultural Sciences, Bangalore, India.

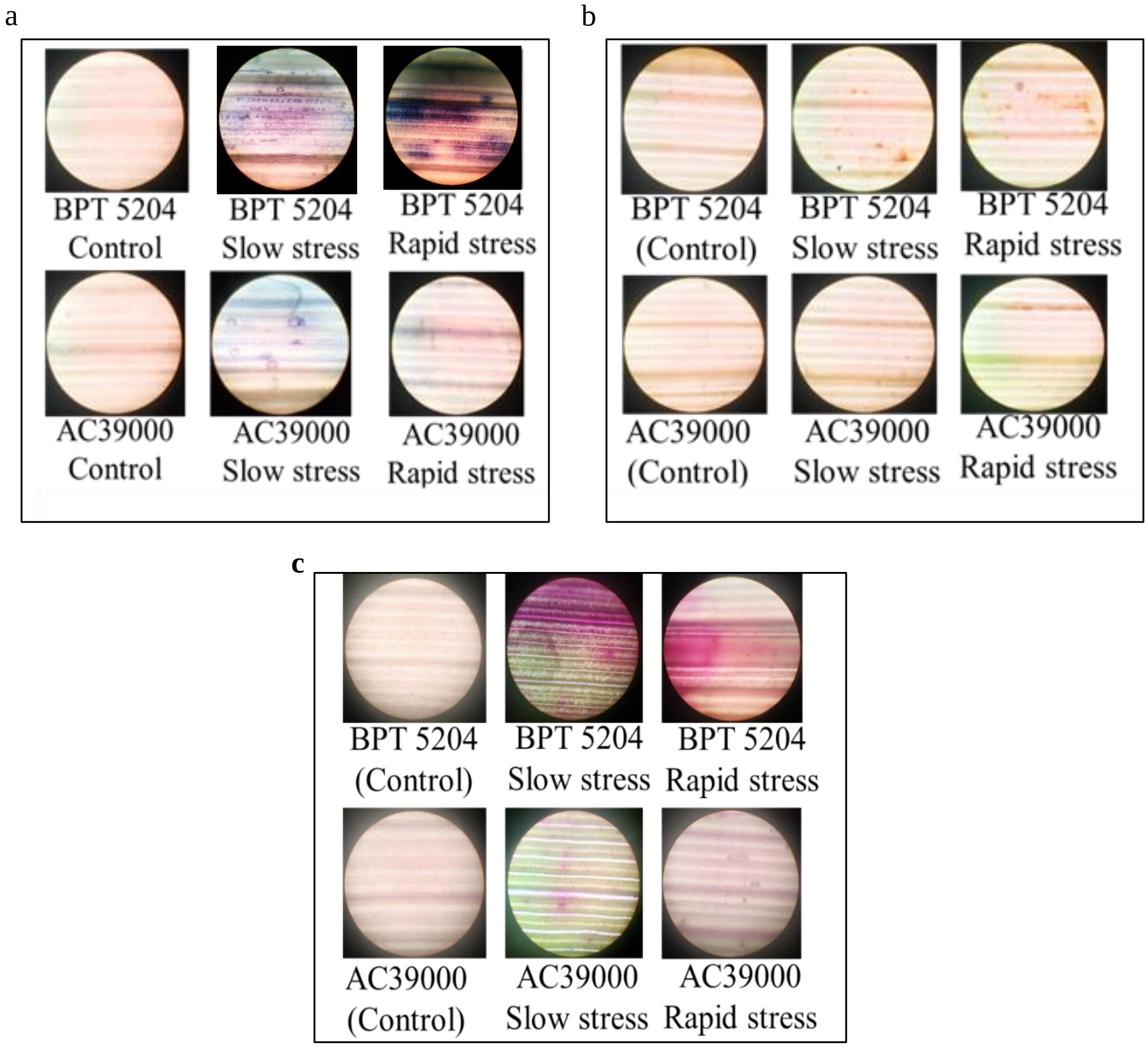

Figure S2: Visualization of oxidative stress biomarkers production in two rice genotypes, BPT 5204, and AC 39000 under control, gradual stress (GS), and rapid stress (RS): ROS production in terms of (a) superoxide content using NBT staining assay and (b) hydrogen peroxide content using DAB staining. (c) RCCs production using schiff base staining assay.

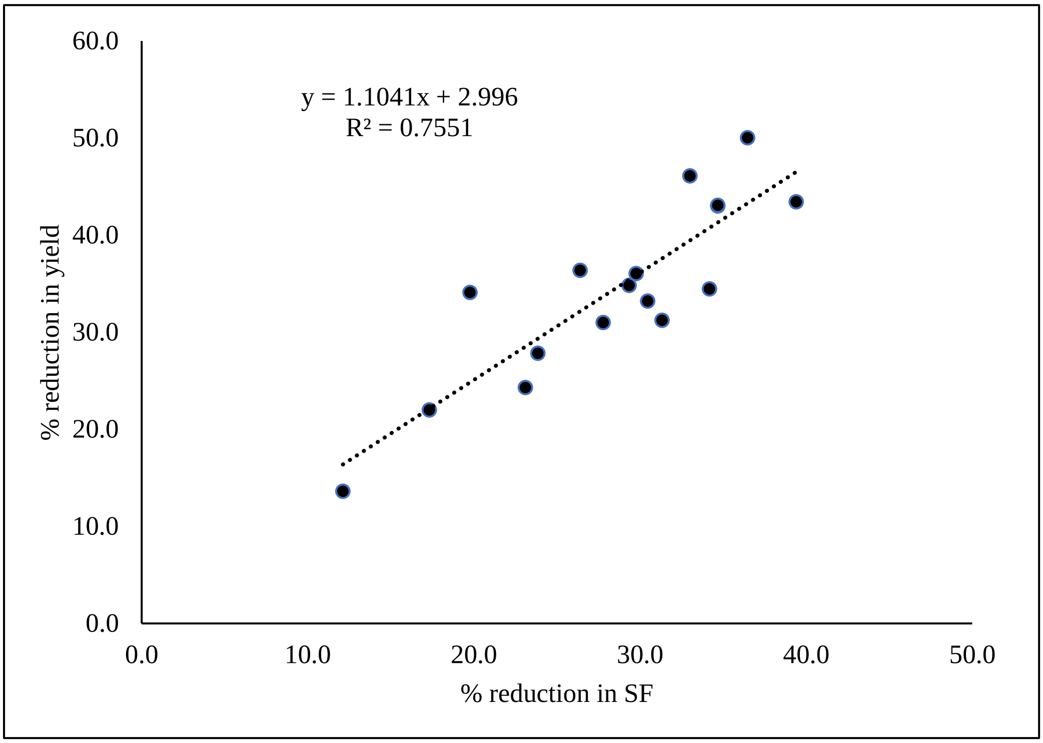

Figure S3: Correlation graph between percent reduction in yield and spikelet fertility (SF) in 17 germplasm lines.
