# Supplementary material for: Induction of Acquired Tolerance Through Gradual Progression of Drought Is the Key for Maintenance of Spikelet Fertility and Yield in Rice Under Semi-irrigated Aerobic Conditions
Source: Front Plant Sci. 2021 Feb 18;11:632919. doi: 10.3389/fpls.2020.632919 (PMC7930615; doi:10.3389/fpls.2020.632919)
Supplement: Supplementary file 2 [file Data_Sheet_2.pdf]

Table S1: Details of primers used for Quantitative RT-PCR

Name of the gene	Primer sequence	Amplicon size	Reference
APX	F: 5′- AGTGTGAACCAGCAGACTAC -3′<br>R: 5′- TCAAGACAGTAGCAGGGGAA -3′	198	Yoo et al., 2017
CAT	F: 5′- AAGGCCAGACAATGTCAGAT -3′<br>R: 5′- GTGGCATTAATACGCCAGTA -3′	203	Yoo et al., 2017
Fe-SOD	F: 5′- TGCACTTGGTGATATTCCACTC-3′<br>R: 5′- CGAATCTCAGCATCAGGTATCA-3′	297	Çelik et al., 2019
ADH1	F: 5′ -GCCATGAAGCTGGAGGTAT-3′<br>R: 5′ -GAAGTCCCGACGAAATGGTA-3′	238	Rauf et al., 2019

Table S2: List of 17 germplasm lines screened to assess genetic variability in acquired tolerance traits at reproductive stage

Genotype number	Genotype name
GEN 32	KABERI::IRGC 66801-1
GEN 224	UPRH 166::IRGC 61615-1
GEN 238	GORA DHAN 2::IRGC 66269-1
GEN 289	ARC 10894::IRGC 21122-1
GEN 346	ARC 7056::IRGC 40914-1
GEN 413	CUTTACK 29::IRGC 49573-1
GEN 443	ANENOE::IRGC 54140-1
GEN 447	PORONG::IRGC 76983-1
GEN 800	MONMINEUPLEU::IRGC 50873-1
GEN 484	RAMADITRA::IRGC 97124-1
GEN 498	MHARAKA::IRGC 70612-1
GEN 541	C 166-135::IRGC 50633-1
GEN 556	IR 19058-107-1::IRGC 72997-1
GEN 584	YAHNG LEU::IRGC 78279-1
GEN 643	DOK HIEN NOI::IRGC 107021-1
GEN 670	PULUTAN::IRGC 71596-1
GEN 769	CHAN THANH HOA::IRGC 60647-1

Table S3: Comparison of the production of ROS (O_2^-, H_2O_2) and RCCs (malondialdehyde (MDA)) among 17 rice germplasm lines.

**Genotypes**	**O_2^- content (Absor × 1000)**			**H_2O_2 content (Absor × 1000)**			**MDA (µg g^{-1}FW)**		
	Control	Stress	Percent change	Control	Stress	Percent change	Control	Stress	Percent change
**GEN 32**	84.9±3.9	155.0±5.6	45.2±4.9	80.0±7.8	106.5±5.5	24.9±2.8	61.45±1.1	74.74±2.3	17.8±2.3
**GEN 224**	338.0±9.9	379.0±18.4	10.8±1.7	85.5±2.5	93.0±5.0	8.1±0.6	56.77±1.2	58.64±1.8	5.0±0.7
**GEN 238**	262.0±15.1	385.0±12.1	31.9±2.6	123.2±4.6	205.5±2.1	40.1±2.0	53.03±2.8	59.39±0.6	10.7±0.6
**GEN 289**	386.5±16.3	761.0±23.7	49.2±5.7	202.0±1.4	383.3±3.9	47.3±0.6	53.08±0.1	64.25±4.9	17.4±1.9
**GEN 346**	225.5±3.5	405.7±12.0	44.4±1.9	180.5±3.5	304.9±4.4	40.8±0.2	48.05±2.9	60.29±4.5	20.3±2.5
**GEN 413**	505.0±2.8	738.5±14.5	31.6±4.5	151.5±2.1	193.0±9.9	21.5±2.1	54.87±2.2	71.85±5.8	21.8±2.8
**GEN 443**	308.3±10.5	600.0±14.1	48.6±0.1	81.0±0.2	130.0±0.0	37.7±0.0	58.45±4.5	70.8±4.5	20.3±3.5
**GEN 447**	278.0±8.5	919.0±44.7	69.7±3.9	106.0±9.9	170.0±2.9	45.6±3.4	58.97±1.9	89.31±0.5	34.0±0.5
**GEN 484**	372.3±9.6	861.0±7.0	56.8±0.9	145.3±5.9	387.7±10.2	62.5±1.0	59.9±7.4	87.88±5.4	31.8±3.4
**GEN 498**	520.7±15.31	983.8±34.7	47.1±3.0	98.4±2.9	140.0±8.9	29.7±3.3	63.08±3.2	90.35±0.6	30.2±0.6
**GEN 541**	343.3±5.5	484.3±17.9	27.6±1.9	135.0±9.9	153.0±9.9	11.8±0.5	58.27±0.9	62.94±0.6	7.4±0.6
**GEN 556**	327.7±11.6	757.5±39.9	56.7±3.6	94.3±2.1	164.0±4.1	42.5±3.3	56.77±2.8	74.73±3.7	24.0±3.7
**GEN 584**	669.5±36.1	1193.0±57.7	41.4±1.1	297.5±6.4	695.0±7.1	57.2±0.9	97.28±3.8	113.02±2.9	13.9±0.9
**GEN 643**	586.5±2.1	1190.5±51.6	48.6±2.3	152.5±7.7	299.5±6.4	49.1±4.8	53.97±0.9	69.02±1.8	21.8±1.8
**GEN 670**	412.0±10	1003.5±57.3	58.9±1.6	130.0±6.9	321.7±8.6	54.9±4.4	54.15±3.4	69.93±2.6	22.6±2.6
**GEN 769**	154.0±15.6	255.5±37.4	39.7±5.2	46.7±4.0	97.7±7.0	45.6±3.6	46.98±3.3	63.13±5.9	25.6±2.9
**GEN 800**	427.0±15.6	777.0±44.9	45±4.0	146.0±8.5	238.7±7.0	38.8±6.6	55.65±3.3	70.05±5.2	20.6±1.2

Table S4: Comparison of the production of membrane damage, total antioxidant capacity (TAC) and total proline among 17 rice germplasm lines.

**Genotypes**	**Membrane damage (ng Evans blue g^{-1}FW)**			**Total antioxidant capacity (mg TAE g^{-1} extract)**			**Total proline (µg g^{-1}FW)**		
	Control	Stress	Percent change	Control	Stress	Percent change	Control	Stress	Percent change
**GEN 32**	7.5±0.5	13.9±1.1	46.4±5.1	1.9±0.0	2.48±0.1	24.8±1.1	4.3±0.3	9.0±0.3	52.4±0.8
**GEN 224**	10.6±0.2	11.1±0.4	3.9±0.2	2.1±0.0	3.68±0.1	43.6±0.7	3.4±0.8	7.1±0.8	39.1±0.6
**GEN 238**	15.0±1.7	18.5±0.0	19.0±0.9	1.9±0.1	2.87±0.1	34.1±0.4	1.7±0.3	7.6±0.3	77.5±1.1
**GEN 289**	14.6±1.4	21.3±1.5	31.5±2.2	2.9±0.0	4.13±0.1	30.0±1.7	9.3±0.6	11.6±0.6	20.1±1.1
**GEN 346**	9.7±0.6	11.9±1.2	18.5±1.1	2.3±0.0	2.45±0.2	4.9±0.5	4.4±0.8	11.1±0.8	60.6±0.6
**GEN 413**	17.7±0.7	22.4±0.2	21.1±2.3	1.6±0.1	3.64±0.1	54.8±2.0	3.9±0.1	18.7±0.1	79.4±2.4
**GEN 443**	8.2±0.9	12.7±1.2	35.3±2.6	2.4±0.0	2.76±0.1	13.8±0.9	7.0±0.8	7.9±0.8	11.2±0.0
**GEN 447**	11.9±0.8	24.7±1.8	51.6±1.2	1.9±0.1	2.71±0.1	29.9±1.6	4.4±0.9	7.3±0.9	40.4±0.9
**GEN 484**	13.4±0.6	30.6±0.2	56.3±1.3	3.5±0.1	4.03±0.0	14.3±0.5	11.3±0.9	20.8±0.9	45.9±0.3
**GEN 498**	23.0±1.1	38.4±2.9	40.1±2.2	2.7±0.1	2.95±0.3	8.7±0.1	8.1±0.9	25.3±0.9	68.0±3.1
**GEN 541**	8.8±0.8	10.1±0.4	12.9±0.7	1.4±0.0	2.27±0.2	38.8±2.8	4.3±0.4	5.6±0.4	22.4±1.4
**GEN 556**	15.6±1.4	29.7±0.6	47.4±1.5	2.1±0.0	2.83±0.0	25.7±1.2	6.5±0.6	19.7±0.6	66.9±0.9
**GEN 584**	31.8±0.1	46.8±1.6	32.0±1.6	6.8±0.0	7.39±0.2	7.6±0.2	7.1±0.3	8.1±0.8	11.7±0.0
**GEN 643**	13.4±0.4	19.2±0.2	30.1±2.1	2.7±0.2	3.92±0.1	31.5±0.5	5.6±0.5	8.4±0.5	33.2±0.3
**GEN 670**	18.8±0.2	37.3±0.8	49.5±1.2	2.8±0.1	3.99±0.0	30.9±1.7	6.2±0.6	10.7±0.6	41.8±0.5
**GEN 769**	7.6±1.6	10.6±0.3	27.9±0.4	1.8±0.1	2.4±0.0	26.0±1.1	7.0±0.9	13.6±0.9	48.2±0.2
**GEN 800**	22.9±1.7	37.9±0.3	39.6±1.2	2.2±0.0	3.61±0.3	40.4±2.1	5.5±0.3	6.4±0.3	13.9±0.3

Table S5: Comparison of total dry matter production (TDM, g pot[1]), spikelet fertility (SF%) and yield (g pot^{-1}) among 17 rice germplasm lines

Genotype	TDM (g pot^{-1})			SF (%)			Yield (g pot^{-1})		
	Control	Stress	Percent change	Control	Stress	Percent change	Control	Stress	Percent change
**GEN 32**	167.9±10.3	137.6±8.8	18.1±0.3	91.7±3.7	64.4±2.4	29.8±4.1	108±19.4	69.1±2.4	36.1±4.3
**GEN 224**	220.3±13.2	200.7±0.4	8.9±0.6	93.4±1.0	77.2±3.8	17.3±3.8	135.5±7.3	105.7±2.0	22.0±2.9
**GEN 238**	236.4±16.9	213.7±7.6	9.6±0.5	87.2±4.7	67.0±1.9	23.1±2.1	128.2±12.9	97.1±2.8	24.3±3.7
**GEN 289**	278.2±13.1	217.3±8.8	21.9±1.3	97.5±1.4	67.8±4.1	30.4±4.7	130±4.0	86.8±4.9	33.2±4.1
**GEN 346**	210.4±5.5	152.6±3.6	27.5±3.3	93.4±2.4	62.5±0.3	33.0±1.3	123.3±7.2	66.4±10.8	46.1±5.4
**GEN 413**	217.5±12.9	215.4±2.2	1.0±0.0	84.0±4.1	67.4±4.0	19.8±0.9	132.9±12.2	87.6±2.2	34.1±2.3
**GEN 443**	194.8±11.7	141.3±1.0	27.5±2.6	95.4±1.2	60.6±5.2	36.5±7.2	86.3±14.3	43.1±1.0	50.0±4.7
**GEN 447**	187.7±11.2	138.8±1.8	26.1±2.6	93.7±0.7	61.2±4.5	34.7±4.2	119.1±3.9	67.8±8.9	43.1±5.8
**GEN 484**	215.0±9.6	198.6±18.6	7.6±0.1	92.5±6.5	56.0±1.7	39.4±1.7	105.6±7.0	59.7±4.1	43.4±1.4
**GEN 498**	184.6±9.2	148.9±4.9	19.3±1.1	95.2±2.5	62.7±0.4	34.2±1.5	107.6±11.6	70.5±5.2	34.5±1.7
**GEN 541**	232.2±8.6	217.3±9.8	6.4±0.03	91.8±4.1	80.7±2.9	12.1±4.3	123.6±3.4	106.8±3.2	13.6±1.9
**GEN 556**	222.2±11.0	171.0±1.8	23.0±3.1	71.9±0.2	47.0±3.5	34.7±4.9	133.3±8.7	76.0±3.1	43.0±7.9
**GEN 584**	185.9±7.4	149.3±10.3	19.7±1.8	87.2±2.6	63.0±3.4	27.8±4.9	102.4±3.6	70.6±1.8	31.0±4.1
**GEN 643**	229.2±7.4	198.7±9.3	13.3±2.1	76.9±1.3	52.8±4.8	31.3±6.4	106.9±2.3	73.5±2.0	31.2±2.7
**GEN 670**	243.3±10.6	206.3±13.1	15.2±1.7	95.0±1.7	67.2±4.9	29.3±4.5	118±10.8	76.9±10.9	34.8±2.6
**GEN 769**	203.3±6.6	168.7±3.3	17.0±1.3	85.2±1.9	62.7±1.9	26.4±3.2	115.7±7.6	73.6±7.4	36.4±5.7
**GEN 800**	223.7±10.7	195.0±11.9	12.9±1.1	91.4±2.4	69.6±1.3	23.8±0.6	110.8±9.9	80.0±3.6	27.8±4.7
